# OA15 Separating the signal from the noise: multiple diagnoses of infection complicating a case of ANCA-associated vasculitis

**DOI:** 10.1093/rap/rkae117.015

**Published:** 2024-11-05

**Authors:** Nicola Heyer, Raminder Aul

**Affiliations:** St. George’s University Hospital, London, United Kingdom; St. George’s University Hospital, London, United Kingdom

## Abstract

**Introduction:**

Infection plays a significant role in rheumatological practice in a variety of different ways. It can be the underlying aetiology of a rheumatological presentation, a key differential to rheumatological disease, or complicate autoimmune disease. Our case describes a patient presenting with cavitating lung disease whose journey to a diagnosis of ANCA-associated vasculitis (AAV) was complicated by diagnostic momentum focusing on infective aetiologies, as well as concomitant influenza A infection and potential non-invasive pulmonary aspergillosis.

**Case description:**

A 67-year-old male presented with 4-weeks of cough, breathlessness and malaise, but no fevers, weight-loss or night sweats. His symptoms started during a 6-week trip to India where he was admitted and treated for community-acquired pneumonia. Historically, he was treated for latent tuberculosis (TB) with rifampicin and isoniazid, although compliance was unclear.

On presentation, he was hypoxic with crepitations and wheeze on chest auscultation. His inflammatory markers were raised (CRP 193 mg/L; WCC 17.3x10*9/L), but renal function was normal. A CXR showed right upper lobe cavitation, with CTPA demonstrating bilateral florid patchy peribronchial opacification with multiple upper lobe cavities.

Broad-spectrum antibiotics were initiated covering for multi-drug resistant organisms with initial improvement in his inflammatory markers. Multiple blood cultures were negative. Acid-fast bacilli smears and TB PCR were negative, but due to increasing oxygen requirements, TB treatment was started. Following a positive beta-D-glucan (>110pg/mL), amphotericin B was initiated for possible pulmonary aspergillosis. He contracted influenza A and deteriorated progressing to type 1 respiratory failure requiring intubation and admission to intensive care, with worsening lung changes on CT-CAP. TB treatment was stopped after multiple negative TB cultures. A bronchoalveolar lavage was galactomannan positive, fuelling concern over pulmonary aspergillosis.

Lack of clinical improvement after a month and worsening renal function led to an ILD referral and the possibility of vasculitis was raised. An autoimmune screen was sent with a positive p-ANCA and PR3 antibody (89.1 IU/mL), and raised urine protein-creatinine ratio (778 mg/dL), securing a diagnosis of AAV.

Treatment induction was started with intravenous cyclophosphamide (CYCLOPS regimen) and pulsed intravenous methylprednisolone with a prednisolone weaning regime. Treatment for pulmonary aspergillosis was continued but switched to anidulafungin due to interaction with cyclophosphamide. He had an excellent response to cyclophosphamide with normalisation of his inflammatory markers, improvement in radiology and successful discharge home one month later.

**Discussion:**

This case illustrates a common clinical conundrum in rheumatology; distinguishing active rheumatological disease from infection. Ensuring the correct diagnosis is imperative to facilitate appropriate treatment, as the consequences of starting incorrect treatment are potentially disastrous.

Given this patient’s travel and medical history, infection, namely TB, was at the forefront of his diagnostic and therapeutic journey. The initial CXR showed a right upper lobe cavity consistent with TB. This, together with the patient being admitted under infectious diseases contributed to confirmation bias and diagnostic momentum in pursuit of an infective diagnosis. Autoimmune disease was only considered one month into admission after further clinical deterioration.

‘Regression to the mean’ refers to extreme results tending to the average upon subsequent measurements. This case highlights the need for caution when attributing a change to an intervention. While there was superimposed viral and fungal infection, improvement in inflammatory markers when starting antibiotics was likely instead due to this phenomenon, contributing to diagnostic bias. It was only when immunosuppression was started that he had a clear clinical and biochemical response.

Differentiating tuberculosis from isolated pulmonary vasculitis is a challenge. This is compounded by the long time-to-result for tuberculosis cultures. Evidence of other organ involvement, particularly renal disease, should be sought. This patient’s declining renal function was attributed to severe illness and wasn’t investigated. Nephrotic-range proteinuria was only identified when his immunology was positive. Had this been investigated at onset, it may have facilitated earlier diagnosis and reduced hospital stay.

Finally, while our patient’s underlying diagnosis was vasculitis, his admission was complicated by superimposed infection. The influenza infection was likely exacerbated by vasculitic lung changes, and immunoparalysis from underlying autoimmune disease. While invasive pulmonary aspergillosis typically occurs with severe neutropenia, risk factors for non-invasive disease are non-specific, including ICU admission, chronic respiratory disease and recent influenza infection.

**Key learning points:**

• Infection is an important part of the differential for AAV, with TB being a key consideration in pulmonary presentations. While negative acid-fast bacillus smears and TB PCR have good specificity, multiple TB cultures are the gold-standard and only test with sufficient sensitivity to rule out the diagnosis. When initiating a treatment, such as antibiotics, care should be taken to avoid overly attributing improvement in inflammatory markers to the intervention, particularly if there is a plateau, or if the clinical picture is not concomitantly improving.

• If the differential is between pulmonary TB and isolated pulmonary AAV, it is important to remember that single-organ disease in AAV is atypical. Additional organ involvement should be actively sought with history, examination and screening investigations. Renal disease in AAV is most commonly asymptomatic, with an active urinary sediment and variable degrees of renal dysfunction. Normal renal function in a renally asymptomatic patient does not rule out renal involvement. A urine protein-creatinine ratio should therefore be completed regardless of renal function.

• Atypical ANCA serology can be associated with infection and inflammatory bowel disease, however, it does not rule AAV out of the differential. Biopsy of the involved organ is the gold-standard in diagnosis and can be confirmatory where there is concern about a false positive result. This patient was too clinically unstable to have a lung or renal biopsy.

• In the context of autoimmune disease, infection is often linked to immunosuppression from treatment. However, infection can also occur with active disease due to immunoparalysis and altered organ structure and function. Underlying chronic respiratory disease, such as pulmonary vasculitis, is a risk factor for non-invasive pulmonary aspergillosis. Finally, dual treatment of pulmonary fungal disease and vasculitis may be complicated by interactions between treatments, such as between cyclophosphamide and amphotericin B.

